# Supplementary material for: “What do you mean I can’t have a doctor? this is Canada!” – a qualitative study of the myriad consequences for unattached patients awaiting primary care attachment
Source: BMC Prim Care. 2022 Mar 30;23:60. doi: 10.1186/s12875-022-01671-5 (PMC8966849; doi:10.1186/s12875-022-01671-5)
Supplement: Supplementary file 1 — Additional file 1. [file 12875_2022_1671_MOESM1_ESM.pdf]

nova scotia health authority

Unattached Patient Interview Guide – UP-study
For telephone or in-person interviews

Introduction

First off, I want to thank you for taking the time to speak with me today! Your participation in our study is greatly appreciated. My name is ___________________ and I am a researcher from [Institution], working under the supervision of [PI's name]. I am here to talk to you today about your experiences with health care, and in particular, your experiences related to not having a regular primary health care provider such as a family doctor.

Confirm Informed Consent

- You were given a consent form that you were asked to review and sign. The consent form contained information about the study and how the information you provide will be used. At this point, do you have any questions about the consent form or any of the information you received?

- Your participation in this interview is entirely voluntary. You are free to withdraw your consent to participate at any time before, during or up to four weeks after the interview. In this case, we will not use any information you have provided us. You may also choose to not answer any of the questions.

- Before we begin, I wanted to give you some information regarding your participation as well as some background information and the purpose of our study.

Background Information

- Today we will be talking about access to primary health care, in particular access to care with family doctors, nurse practitioners and other places you might seek health care. We use the term 'primary health care' to refer the typical health care providers people seek when they are first sick or have questions about their health. This is most often a family doctor, but can also be a nurse practitioner, pharmacist and other health professional.

- The purpose of our study is to understand more about what we call "unattached patients", meaning people who do not have a regular primary health care provider to go to for care.

- **VERIFY THEY DID/DO NOT HAVE A PRIMARY CARE PROVIDER (family physician or nurse practitioner) at this point, or have at least recently been in this situation.**

- This is part of a larger study that aims to understand more about "unattached patients", how they became unattached, and the consequences of being unattached, including for their health and health care. In another part of this research, we are also talking with health care providers and health policy makers to see what can be done to try and reduce the number of unattached patients.

- You have been invited to participate in this study because your experiences will help identify the issues important to unattached patients, the consequences of unattachment for you and the system. The information you provide through this study will help guide recommendations for health care providers and the health care system about how to improve access to primary health care in our province.

- As someone with lived experience of not being able to find a primary health care provider, you are an expert on this subject, and I am asking you to share that expertise. I would like to ask you questions about your health and health care experiences, and your views on the importance of having a regular health care provider whose should help you with this, and what factors are most important to you.

- Do you have any questions before we begin?

- The interview will be audio recorded so we can be sure we capture your input accurately and so that we can combine the information we get from you with what we learn from other unattached patients. What you share with us will remain anonymous. We may use quotes from your interview for research purposes, but your name will never appear in any reports, and your responses will be presented in a way that they cannot be traced back to you. Is it ok if I begin recording?

- **START AUDIO RECORDING**

ABOUT YOUR HEALTH CARE

1. Have you previously had a regular provider/family physician? Can you tell me a bit about that?

2. Please describe how you came to be in need of a regular provider/family physician?
    a. Optional Probes: When did that begin? Can you tell me more about….
    b. How would you describe the impact of not having a regular provider?
    c. Do you have any health conditions that you feel require a the care of regular primary health care provider (PHCP)?

3. What steps have you taken to find a regular provider?
    a. Optional probes: have you called anyone? The Primary Health Care Connects Service or equivalent in other zones? Registered with 811? Inquiring a local family practices? Through friends and family? Social media?

4. What other health care services have you used during this time of not having a regular provider?
    a. Probe: Walk in clinics, ED, pharmacy, alternative/complementary medicine such as acupuncture, naturopath etc.

5. Have you had any expenses related to not having a regular provider?
    a. Probes: Costs associated with other allied health professionals? Fees charged by alternative care providers? Child care and/or work time lost while waiting in ED or at walk in clinics? Travel costs to get to ED or walk in clinics? Over the counter medications to supplement prescriptions?

6. How would you say your health has been during this time?
    a. Do you think your health has been affected by not having a regular PHCP?
    b. Can you describe how your health has improved or deteriorated?

7. Please tell me about any informal supports for your health or health care such as family members or friends?
    a. How do the help you?

8. Can you describe to me a 'best-case scenario' in terms of how a primary health care provider - or primary health care team - might meet your health care needs?

Closing

Is there anything we have not talked about that you believe is important to understand about being an unattached patient? If so, what?

Probes:

- What do you mean when you say X?
- I'm not sure I understand X, will you explain that to me?
- Can you give me an example of X?

- Why do you think X?
- How did this/that happen/come to be?
- What happens then? / What do you do then?

- Can you tell me more about that?
- Can you elaborate on that?

- How do you handle X?
- How does X affect you?
- How did/do you feel about X?

- Can I go back to X for a second?
